# Hip surgery and its evidence base: progress over a decade?

**DOI:** 10.1007/s10195-016-0421-z

**Published:** 2016-07-21

**Authors:** Kamrul Hasan, Shivakumar Shankar, Aadhar Sharma, Alison Carter, Razi Zaidi, Suzie Cro, John Skinner, Andy Goldberg

**Affiliations:** 1 Whipps Cross University Hospital, Whipps Cross Road, London, E11 1NR UK; 2 Royal National Orthopaedic Hospital, Brockley Hill, Stanmore, Middlesex HA7 4LP UK; 3 Barnet General Hospital, Wellhouse Lane, Barnet, Hertfordshire EN5 3DJ UK; 4 Royal National Throat, Nose and Ear Hospital, Grays Inn Road, London, WC1X 8DA UK; 5 MRC Clinical Trials Unit, London, WC2B 6NH UK

**Keywords:** Evidence-based medicine, Hip, Arthroplasty

## Abstract

**Background:**

The burden of traumatic and elective hip surgery is set to grow. With an increasing number of techniques and implants against the background of an aging population, the emphasis on evidence-based treatment has never been greater. The purpose of this study was to assess changes in the levels of evidence in the hip literature over a decade.

**Materials and methods:**

Articles pertaining to hip surgery from the years 2000 and 2010 in *Hip International*, *Journal of Arthroplasty*, *Journal of Bone and Joint Surgery* and *The Bone and Joint Journal* were analysed. Articles were ranked by a five-point level of evidence scale and by type of study, according to guidelines from the Centre for Evidence-based Medicine.

**Results:**

531 articles were analysed from 48 countries. The kappa value for the inter-observer reliability showed excellent agreement between the reviewers for study type (*κ* = 0.956, *P* < 0.01) and for levels of evidence (*κ* = 0.772, *P* < 0.01). Between 2000 and 2010, the overall percentage of high-level evidence (levels I and II) studies more than doubled (12 to 31 %, *P* < 0.001). The most frequent study type was therapeutic; the USA and UK were the largest producers of published work in these journals, with contributions from other countries increasing markedly over the decade.

**Conclusions:**

There has been a significant increase in high levels of evidence in hip surgery over a decade (*P* < 0.001). We recommend that all orthopaedic journals consider implementing compulsory declaration by authors of the level of evidence to help enhance quality of evidence.

**Level of evidence:**

Level 2: economic and decision analysis.

## Introduction

More than 1.7 million hip replacements were performed across the globe in 2013; this figure is expected to increase to 2.5 million by 2020 [1]. Furthermore, a recent international survey of 291 conditions has found hip and knee osteoarthritis to be the 11th highest contributor to global disability [2]. A steady rise in demand combined with a global trend towards financial austerity and the subsequent increased pressures on health care providers has contributed to an increased emphasis on treatments underpinned by a strong evidence base. This enables clinicians to optimise patient outcomes while simultaneously demonstrating value for service. With a seemingly endless variety of new and emerging surgical innovations and technologies, it remains the responsibility of the orthopaedic community to produce evidence to support the best practice in their field.

The assembly of an experienced Orthopaedic Data Evaluation Panel (ODEP), whose role is to examine the evidence either supporting or refuting the use of certain implants and to apply a grading system depending on their performance, only supports the notion that high-quality evidence is having an ever greater influence on the usage of orthopaedic implants and is only set to increase in the future [3].

The basic principle of evidence-based medicine (EBM) is that we should treat where there is evidence of benefit and not treat where there is evidence of no benefit (or harm) [4]. Higher level of evidence studies serve to produce treatment that is efficacious and cost-effective [5].

The Oxford Centre for Evidence-based Medicine (CEBM) classifies research into four main types: therapeutic, prognostic, diagnostic and economic. These are further sub-classified into five levels: I (high) to V (low) [6]. The *Journal of Bone and Joint Surgery* (*JBJS*) introduced a section devoted to the promotion and dissemination of high-quality evidence in the year 2000 with randomised controlled trials (level I) forming the main contribution [7]. The method of grading of level of evidence used by the *JBJS* is the same used by the authors in this study and is in accordance with the CEBM system.

The *JBJS* also notably introduced declarations of levels of evidence for research articles at the time of publication in January 2003; this practice is on the rise and looks set to continue. Furthermore, studies have shown that the grading system introduced by the CEBM can be reliably applied and that clinical investigators should pursue studies with a higher level of evidence whenever feasible [8, 9].

While recently published studies have shown some improvements in the quality of published orthopaedic research, the consensus decision is that the overall level of evidence remains low [10]. Considering the size of hip surgery as a sub-specialty, it is perhaps surprising to find that there are no studies devoted to analysing the trend of quality evidence in this field. This is something we aim to address.

The aim of the current study was to investigate the trends of levels of evidence in hip surgery in the year 2000 and again 10 years later in 2010. The study aimed to analyse changes in the quantity of published evidence, the quality and type of this evidence (as per the CEBM), geographical variations in the origin of published studies and the inter-observer agreement of the classification of the level of evidence among the reviewers.

## Materials and methods

Articles published in hip surgery journals in two specific years, 2000 and 2010, were analysed. The study intervals were from January 1, 2000 to December 31, 2000 and from January 1, 2010 to December 31, 2010.

We included four well recognised journals that were affiliated to orthopaedic and hip societies. The journals had to be in the English language, and must have published in print and online for the entire period of study. We included *Hip International*, affiliated to the European Hip Society; the *Journal of Arthroplasty*, affiliated to the American Association of Hip and Knee surgery; the *Journal of Bone and Joint Surgery* (*JBJS*), affiliated to the American Academy of Orthopaedic Surgeons (AAOS); and *The* *Bone and Joint Journal* (*BJJ*), affiliated to the British Orthopaedic Association (BOA). In 2013 the *Journal of Bone and Joint Surgery* (British, or *JBJS-Br*) was renamed the *BJJ*. In this paper we shall refer to it as the *BJJ* despite the fact that it refers to the journal titled the *JBJS-Br* in both time periods captured.

Two independent reviewers (AC and AA) analysed the journals and graded them in line with the system described by the CEBM. Each article type was allocated into therapeutic, prognostic, diagnostic and economic, and level of evidence was assigned on a scale of I–V, with I being considered the strongest level and V the weakest level. Any disagreements were discussed with the senior author (AG) and the methodology described by Spindler et al. was used to reassess such papers before a final decision with regard to the article level or type was made [11]. In this important paper from 2005, the authors commendably described in detail a method for applying EBM when reviewing a manuscript (described below) and therefore applying a level for the published evidence. When deciding on level and type of evidence, it is the methods section of a manuscript that of course proves crucial.

The methods section is carefully analysed to identify a study type (therapeutic, diagnostic, prognostic or decision analysis) and a study design (e.g., randomised controlled trial). From an EBM point of view, it is important to ask whether control groups were included, whether data was prospectively or retrospectively collected or, in the case or a diagnostic study, was the gold standard investigation used? The authors’ attempts to remove bias from their study is also noted: this can be achieved by the use of independent examiners, for example. The size and composition of the patient population is noted and importantly the length of follow-up as well as rates of patients being lost to follow-up. The statistical analysis is important, particularly as some statistical tests are appropriate for answering particular questions while others are not.

The reviewers verified their own levels of evidence against the journal’s level where a level of evidence had been provided by the journal. Case reports with more than three subjects were treated as a case series rather than a case report, thereby upgrading the article from level V to level IV evidence.

Published work that was excluded from the study included all animal, cadaveric and basic science studies as well as editorials, surveys, letters to the editor, technical tips and expert opinions.

Inter-observer agreement was measured using the kappa statistic. Kappa values were assessed using the criteria described by Fleis [12].

Fisher’s exact test was used to compare the proportions of the study types and levels of evidence by year of publication, and to compare the proportions of the study types and levels of evidence by journal. Fisher’s exact test was also used to examine the proportions of the study types and levels of evidence by year of publication within the five different journals. All statistical analysis was performed using Stata/IC version 12.1 (StataCorp, College Station, TX, USA). A *P* value <0.05 was considered statistically significant.

## Results

550 publications were reviewed. 19 papers were excluded as per our criteria, leaving 531 included studies.

The kappa values for the inter-observer reliability of study type were excellent (*κ* = 0.956, *P* < 0.01) and showed good agreement for levels of evidence (*κ* = 0.772, *P* < 0.01).

Therapeutic studies constituted the majority of study type (85.7 %) with economic study types in the minority (1.3 %). Level IV evidence was the most frequent (48.6 %) with level I studies the least frequent (9 %) (Table 1).

**Table 1:** Level of evidence and study type by year of publication

	Year		*P* value
	2000	2010	
Type of study			
Diagnostic	25 (14 %)	13 (4 %)	<0.001
Economic	3 (1 %)	4 (1 %)	0.697
Prognostic	11 (6 %)	20 (6 %)	0.999
Therapeutic	144 (79 %)	311 (89 %)	0.001
Level of evidence			
Level 1	10 (5 %)	38 (11 %)	0.039
Level 2	12 (7 %)	69 (20 %)	<0.001
Level 3	19 (10 %)	35 (10 %)	0.881
Level 4	115 (63 %)	143 (41 %)	<0.001
Level 5	27 (15 %)	63 (18 %)	0.394
Total	*N* = 183	*N* = 348	

From 2000 to 2010 there were statistically significant rises in the number of therapeutic studies, level I and level II studies (Table 1). In the same time frame there were statistically significant drops in the proportion of diagnostic and level IV studies (*P* < 0.01) (Table 1). Between 2000 and 2010, the overall percentage of high-level evidence studies (levels I and II) increased and low-level evidence studies (levels III, IV, and V) decreased (Table 2) (*P* < 0.001).

**Table 2:** Levels of evidence grouped into high (level I and II) and low (level III, IV and V) by year of publication

	Year		*P* value
	2000	2010	
Level of evidence			
High	22 (12 %)	107 (31 %)	<0.001
Low	161 (88 %)	241 (69 %)	
Total	*N* = 183	*N* = 348	

**Fig. 1:**
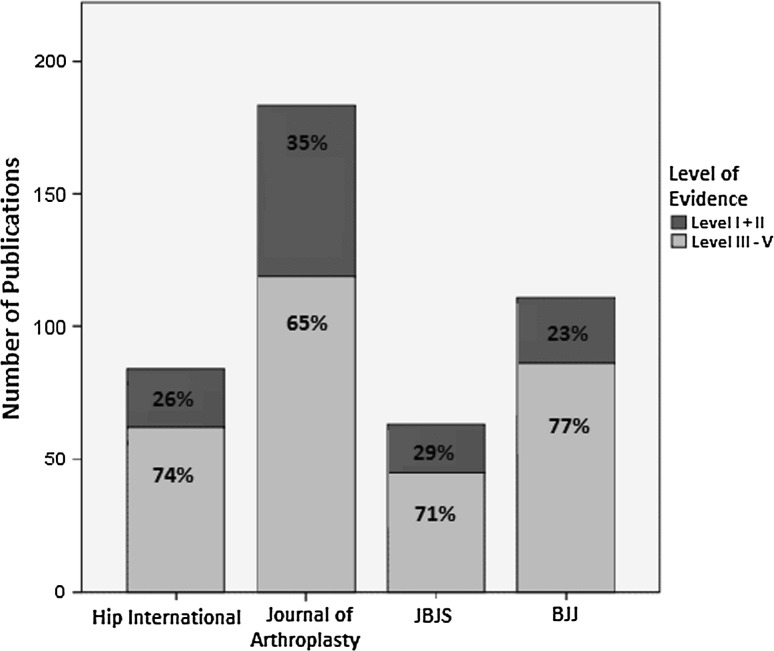
Bar chart illustrating the contribution of each journal to the total volume of hip articles in the combined years 2000 and 2010 as well as each journal’s contribution to both high (I + II) level and low (III–V) level evidence

Within the *Journal of Arthroplasty* and *JBJS* there were significant increases in high-level evidence over the decade. The increase in high-level evidence did not reach statistical significance in *Hip International* or the *BJJ*. The *Journal of Arthroplasty* had the greatest proportion of high-level evidence (35 %) followed by the *JBJS* (29 %), whereas the *BJJ* had the least with 23 % high-level evidence publications (Fig. 1).

**Fig. 2:**
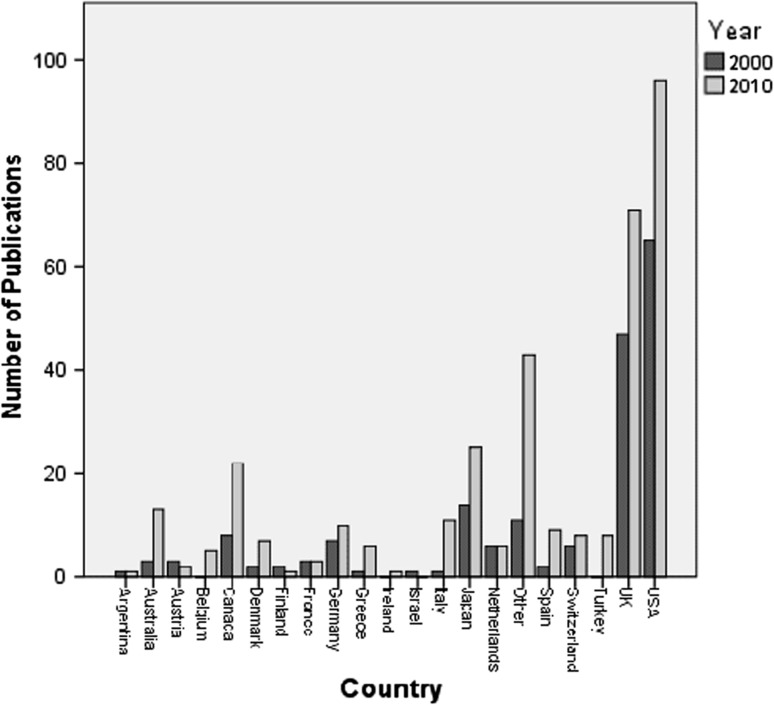
*Bar chart* illustrating the geographical variation of the volume of publications in the included journals by year

A total of 23 countries contributed to the four journals. The total number of papers increased from 183 (in 2000) to 348 (in 2010). The USA and UK were the major contributors in both 2000 (60 %) and 2010 (48 %); however, contributions from outside these countries increased from 40 % in 2000 to 52 % in 2010 (Fig. 2).

## Discussion

The expansion in hip surgery as a sub-speciality is underlined by the explosion in the number of published studies over a decade. The current study is the first to show a statistically significant increase in high-level evidence in the field of hip surgery. We found the occurrence of high-level evidence (I and II) to be 31 %, which compares favourably to other orthopaedic sub-specialties such as foot and ankle, where just 10 % of papers were considered high-level [13], paediatrics, where just 8 % of the published literature was deemed to be high-level [14], and spinal surgery, where 27.9 % of reviewed papers were considered as level I + II [15]. Aside from the foot and ankle literature, which used methodology similar to ours, the other sub-specialty reviews excluded level V studies, which gave a falsely elevated proportion of level I and II papers. We elected to include level V papers in order to establish a truer proportion of high-level studies. We found an excellent level of inter-observer agreement between our reviewers, which is in keeping with previous similar measurements and underlines the reliability of the CEBM system when measuring levels of evidence [9].

It is reassuring that scientific quality of hip-related literature seems to be improving over the last decade. This improvement coincides with an increase in the influence that high-quality evidence is having on the uptake of certain implants and techniques. In the UK this has been overseen by an experienced ODEP made up of senior surgeons and healthcare managers [3]. This has been a busy period for developments in hip surgery, with the rise and fall in popularity of metal-bearing hip arthroplasty components and also hip resurfacing. This has occurred alongside the increased use and acceptance of non-arthroplasty hip surgery including arthroscopic techniques and osteotomies. It is therefore creditable that the highest proportion of published level I and II studies in orthopaedic surgery have been published in the rapidly evolving field of hip surgery compared to any other orthopaedic sub-specialty.

The *JBJS* introduced an author declaration of level of evidence in 2003 [16], which appears to have led to an increased number of level I and II studies in these journals [13, 17].

Epidemiology- and non-epidemiology-trained reviewers can apply the levels-of-evidence guide to published studies with acceptable inter-observer agreement [18]; this is replicated in our study and several other studies reliably to establish the level of evidence.

The vast majority of papers originated from the USA and UK in both 2000 and 2010, although there was an increasing contribution from the rest of the world. This increase in rest-of-world papers might reflect globalisation and an increased tendency to publish in English-language international journals, or in contrast might reflect an editorial policy that is attempting to be more inclusive.

Limitations of this study include detection bias, as the reviewers were not blinded to the journal source. However, the method for inter-observer agreement of level of evidence has been established previously [18]. We acknowledge that we have not included all the orthopaedic journals with hip surgery articles. The inclusion criteria incorporated an affiliation to a major orthopaedic society and the sample falls within the same criteria. We believe that regardless of these limitations our study investigates an exhaustive list of hip surgery articles in four major orthopaedic society journals and for the first time takes into account level V studies and geographical variations in publishing.

We have demonstrated a trend towards higher level of evidence in hip surgery over a decade, with two journals (the *JBJS* and *Journal of Arthroplasty*) showing significant levels of improvement in high-level evidence. We recommend that all orthopaedic journals consider implementing compulsory declaration of the level of evidence by authors at the time of submission to help enhance quality of evidence in our prestigious journals.

## References

[1] Tian L. Is the hip market set for a rebound? | Global data healthcare. http://healthcare.globaldata.com/resources/expert-insights/medical-devices/is-the-hip-market-set-for-a-rebound. Accessed 5 Sept 2014

[2] Cross M, Smith E, Hoy D (2014). The global burden of hip and knee osteoarthritis: estimates from the global burden of disease 2010 study. Ann Rheum Dis.

[3] ODEP statement | ODEP. http://www.odep.org.uk/AboutODEP/ODEPStatement.aspx. Accessed 2 Oct 2015

[4] Belsey J (2009) What is evidence based medicine? Hayward Medical Communications, London pp 1–10

[5] Wenger DR (2012). Limitations of evidence-based medicine: the role of experience and expert opinion. J Pediatr Orthop.

[6] Home | CEBM. http://www.cebm.net/. Accessed 5 Sept 2014

[7] Wright JG, Swiontkowski MF (2000). Introducing a new journal section: evidence-based orthopaedics. JBJS A.

[8] Wright JG, Einhorn TA, Heckman JD (2005). Grades of recommendation. JBJS A.

[9] Obremskey WT, Pappas N, Attallah-Wasif E, Tornetta P, Bhandari M (2005). Level of evidence in orthopaedic journals. J Bone Jt Surg Am.

[10] Carr AJ (2005). Evidence-based orthopaedic surgery: what type of research will best improve clinical practice?. JBJS Br.

[11] Spindler KP, Kuhn JE, Dunn W, Matthews CE, Harrell FE, Dittus RS (2005). Reading and reviewing the orthopaedic literature: a systematic, evidence-based medicine approach. J Am Acad Orthop Surg.

[12] Fleiss J, Levin B, Paik Cho M (2013). Statistical methods for rates and proportions.

[13] Zaidi R, Abbassian A, Cro S (2012). Levels of evidence in foot and ankle surgery literature: progress from 2000 to 2010?. J Bone Jt Surg Am.

[14] Cashin MS, Kelley SP, Douziech JR, Varghese RA, Hamilton QP, Mulpuri K (2011). The levels of evidence in pediatric orthopaedic journals: where are we now?. J Pediatr Orthop.

[15] Amiri AR, Kanesalingam K, Cro S, Casey ATH (2013). Level of evidence of clinical spinal research and its correlation with journal impact factor. Spine J.

[16] Wright JG, Swiontkowski MF, Heckman JD (2003). Introducing levels of evidence to the journal. J Bone Jt Surg Am A.

[17] Wupperman R, Davis R, Obremskey WT (2007). Level of evidence in Spine compared to other orthopedic journals. Spine.

[18] Bhandari M, Swiontkowski MF, Einhorn TA (2004). Interobserver agreement in the application of levels of evidence to scientific papers in the American volume of the Journal of Bone and Joint Surgery. J Bone Jt Surg Am A.

